# Single-Cell RNA Sequencing Reveals Atlas of Yak Testis Cells

**DOI:** 10.3390/ijms24097982

**Published:** 2023-04-28

**Authors:** Xingdong Wang, Jie Pei, Lin Xiong, Shaoke Guo, Mengli Cao, Yandong Kang, Ziqiang Ding, Yongfu La, Chunnian Liang, Ping Yan, Xian Guo

**Affiliations:** 1 Key Laboratory of Yak Breeding Engineering of Gansu Province, Lanzhou Institute of Husbandry and Pharmaceutical Sciences, Chinese Academy of Agricultural Sciences, Lanzhou 730050, China; wxd17339929758@163.com (X.W.); peijie@caas.cn (J.P.); xionglin@caas.cn (L.X.); gsk1125@163.com (S.G.); caomengliaaa@163.com (M.C.); 82101215419@caas.cn (Y.K.); dingziqiang1997@163.com (Z.D.); layongfu@caas.cn (Y.L.); chunnian2006@163.com (C.L.); yanping@caas.cn (P.Y.); 2 Key Laboratory of Animal Genetics and Breeding on Tibetan Plateau, Ministry of Agriculture and Rural Affairs, Lanzhou 730050, China

**Keywords:** scRNA-seq, spermatogenesis, testis, germ cell, somatic cell, yak

## Abstract

Spermatogenesis is a complex process that involves proliferation and differentiation of diploid male germ cells into haploid flagellated sperm and requires intricate interactions between testicular somatic cells and germ cells. The cellular heterogeneity of this process presents a challenge in analyzing the different cell types at various developmental stages. Single-cell RNA sequencing (scRNA-seq) provides a useful tool for exploring cellular heterogeneity. In this study, we performed a comprehensive and unbiased single-cell transcriptomic study of spermatogenesis in sexually mature 4-year-old yak using 10× Genomics scRNA-seq. Our scRNA-seq analysis identified six somatic cell types and various germ cells, including spermatogonial stem cells, spermatogonia, early-spermatocytes, late-spermatocytes, and spermatids in yak testis. Pseudo-timing analysis showed that Leydig and myoid cells originated from common progenitor cells in yaks. Moreover, functional enrichment analysis demonstrated that the top expressed genes in yak testicular somatic cells were significantly enriched in the cAMP signaling pathway, PI3K-Akt signaling pathway, MAPK signaling pathway, and ECM receptor interactions. Throughout the spermatogenesis process, genes related to spermatogenesis, cell differentiation, DNA binding, and ATP binding were expressed. Using immunohistochemical techniques, we identified candidate marker genes for spermatogonial stem cells and Sertoli cells. Our research provides new insights into yak spermatogenesis and the development of various types of cells in the testis, and presents more reliable marker proteins for in vitro culture and identification of yak spermatogonial stem cells in the later stage.

## 1. Introduction

The testis is a vital male reproductive organ composed primarily of spermatogenic tubules and testis mesenchyme [1]. The seminiferous tubules consist of a boundary membrane and a seminiferous epithelium. The latter includes Sertoli cells (SCs) and germ cells, while testis mesenchyme is primarily composed of Leydig cells (LCs), myoid cells (MCs), and macrophages [2]. The testis performs two main functions: exocrine and endocrine [3]. The exocrine function involves the production of highly differentiated gametes, i.e., sperms, which are released into the lumen of the seminiferous tubules and stored in the epididymis [4]. The endocrine function is performed by LCs outside the seminiferous tubules, which secrete androgens, mostly testosterone, to promote gonadal development and spermatogenesis and maintain male secondary sex characteristics [5]. Therefore, the integrity of testicular physiological function is crucial for successful sperm production [6].

Spermatogenesis is a finely regulated process of germ cell proliferation and differentiation occurring continuously in mammals since puberty [4]. The continuous production of male gametes is irreversible after meiosis [7] and depends on the balance between self-renewal and meiosis of spermatogonial stem cells (SSCs). Testicular niche cells play an essential role in guiding the survival and differentiation of male germ cells, and spermatogenesis relies on the complete maturation of the somatic microenvironment [8]. In the adult testis, niche cells, including SCs, LCs, and MCs, provide physical and hormonal support for SSCs to successfully complete spermatogenesis [9]. Therefore, mammalian spermatogenesis is a complex process that requires intricate interactions between germ cells and somatic cells [10].

Spermatogenesis can be divided into three functional stages: mitosis, meiosis, and spermiogenesis. In the final spermatogenesis stage, haploid round spermatids (RS) are transformed into spermatozoa, which are released into the spermatogenic tubule [11] and subsequently enter the epididymis for a series of complex modifications and maturation.

The yak (*Bos grunniens*) is a ruminant that diverged from other ruminants about 2.2 million years ago [12]. Yaks are primarily distributed in the Qinghai Tibet Plateau (QTP) and its adjacent alpine and subalpine regions, where there is no absolute frost-free period and the altitude ranges from 2500 to 6000 m [13]. Yaks have compact body structures, no functional sweat glands, and a relatively small skin surface area per unit of body weight [14]. These characteristics allow yaks to thrive in the alpine hypoxic environment. In China, there are over 16 million yaks, accounting for more than 95% of its global population [15]. Yaks not only provide essential economic products for local herders, such as milk, meat, fur, and fuel [16], but also serve as a means of transportation for the local population [17]. Yaks also play a crucial role in socioeconomic development, pasture ecosystem maintenance, and agricultural biodiversity conservation in the QTP region [18]. However, due to their low reproductive efficiency, delayed puberty, seasonal reproductive mode, low conception rate, and long calving interval [19], yaks generally have lower reproductive performance than other cattle breeds. As testes are vital in male reproduction, further research on yak testes could enhance yaks’ reproductive efficiency. Gong [20] studied piRNA in yak testes at various stages of sexual maturity and identified differentially expressed genes (DEGs) involved in spermatogenesis via the ECM–receptor interaction and PI3K-Akt signaling pathway. Ruan [21] analyzed protein expression profiles of the testis of Tianzhu white yak at different developmental stages, and the expression patterns of regucalcin and heat shock 60 kDa protein in category 2 were confirmed. Wang [22] investigated testes of yaks before and after sexual maturity and examined the methylation-related regulatory enzymes and the overall methylation level of testis tissue. The methylation level of yak testes increased after sexual maturity. However, the presence of multiple germ cell and somatic cell types in the testis [23] makes it challenging to study different cell types at various developmental stages and explore the gene expression of a specific cell type [10].

Since 2009, there has been rapid development in single-cell RNA sequencing (scRNA-seq) platforms [24]. Using this unbiased method, one can sequence thousands of cells in an experiment, obtaining thousands of independent features per cell and providing ultra-high-resolution transcriptomes of animal tissues or organs [25]. Many studies have utilized scRNA-seq to study mammalian spermatogenesis [26,27]. For example, Lukassen [28] sequenced 2500 cells from the testes of two 8-week-old mice by scRNA-seq, capturing the complete continuity of the meiosis and post-meiosis phases of spermatogenesis, making it a well-suited method for marker discovery, network inference, and similar analyses. Lau [7] used scRNA-seq to sequence the transcriptome of cynomolgus macaque testes, and identified the gene characteristics defining the spermatogonia population. The study explored the dynamics of self-renewal and differentiation, and also described in detail the transcriptional changes occurring during the whole process of spermatogenesis. This study highlighted the synergistic activity of DNA damage response (DDR) pathway genes, which have dual roles in maintaining genomic integrity and affecting meiotic sex chromosome inactivation (MSCI). While most previous studies focused on the spermatogenesis of germ cells [29,30], a recent study on the single-cell transcriptome of human testes during puberty revealed the developmental changes of somatic cells [31]. Guo [9] sequenced 6500 testicular cells from adults using scRNA-seq, identified five niche/somatic cell types (LCs, MCs, SCs, endothelial cells (ECs), and macrophages), and observed germ cell–niche interactions and key human–mouse differences.

Previous research has primarily focused on higher primates and model animals, resulting in a need of more information and data on large domestic animals, such as yaks [32,33]. Therefore, in this study, we established the expression profiles of 11,991 cells from sexually mature yak testis using scRNA-seq technology, analyzed the subsets of cells, identified marker genes, and predicted the potential mechanisms of action. These findings offer valuable insights into identifying biomarkers by analyzing cell heterogeneity in yak testis. In addition, the cell lineage inference analysis provides a comprehensive understanding of the molecular pathways determining the fate of major cell lineages, which is essential for future yak breeding endeavors.

## 2. Results

### 2.1. Overview of scRNA-Seq in Yak Testicular Tissue

**Figure 1:**
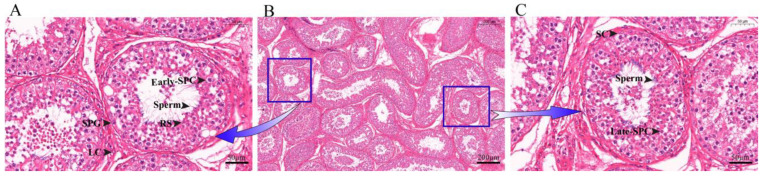
Histological observation of yak testicular tissue sections. (**A**,**C**) 40× magnification. (**B**) 10× magnification. SPG: Spermatogonia; SPC: Spermatocytes; RS: Round spermatids; SC: Sertoli cells; LC: Leydig cells.

To identify the various cell types and marker genes in yak testis, we obtained tissue slices of the organ, which allowed us to observe different types of germ cells and somatic cells (Figure 1A,C). After dissociation, we sequenced 11,991 cells and subsequently filtered 9004 cells according to the specific filtration criteria for further analysis. Each cell had 64,747 reads, with 84.80% of the reads mapped to the yak genome and 77.00% reliably mapped to the genome. The reads of Q30 base in Barcode, RNA Reads, and UMI were 96.90, 90.50, and 96.70%, respectively. A total of 21,035 genes were detected, with an average of about 1004 genes identified in each cell. More information is available in Supplementary Tables S1 and S2.

### 2.2. Cluster Analysis and Cell Type Identification of Testis Cells in Yak

**Figure 2:**
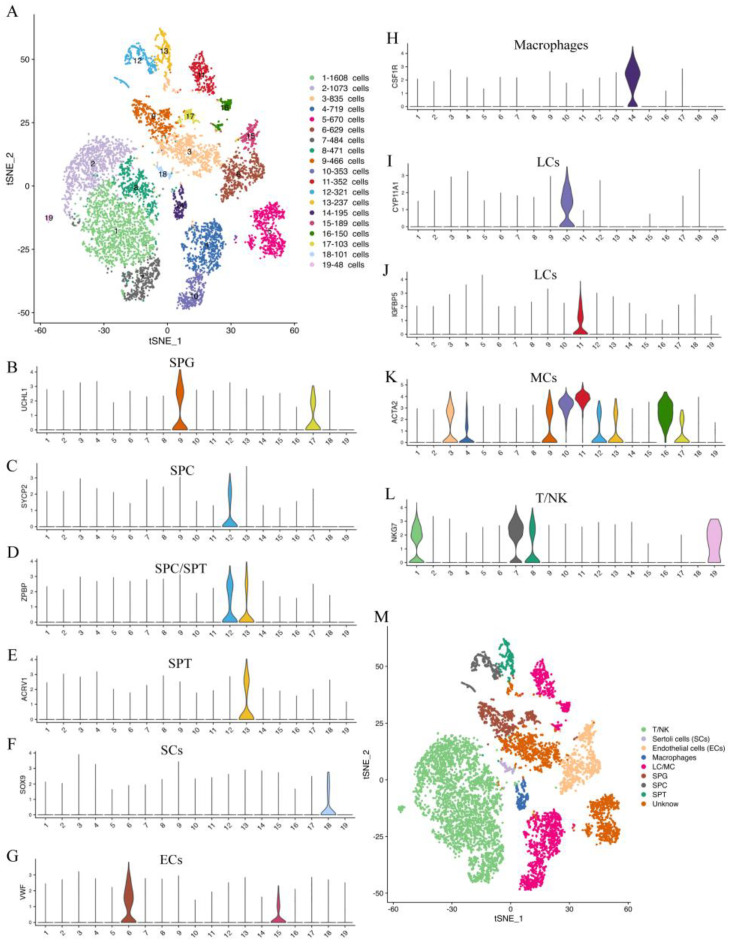
Cluster analysis and cell type identification of yak testicular tissue cells. (**A**) t-distributed stochastic neighbor embedding (tSNE) plots of 10× Genomics profiling of testicular cells from yaks. Each point corresponds to a single cell and is color-coded according to its cluster membership. (**B**–**L**) Violin plots of different cell type-specific gene expression patterns across different clusters. (**M**) The results of cell type identification. Different colors represent different cell types.

In this study, we identified the cell types of each cluster based on the genes expressed in each cluster (Supplementary Table S3) and the known universal marker genes in humans, mice, and other animals. Our findings showed that the testis cells of the yak could be classified into 19 clusters, including 4 germ cell clusters and 13 somatic cell clusters (Figure 2A). The cells in clusters 9 and 17 were found to express the spermatogonia (SPG) marker genes *UCHL1* and *TKTL1* (Figure 2B, Supplementary Figure S1A). The cells in cluster 12 were found to express the spermatocyte (SPC) marker genes *SYCP1*, *MEIOB*, *SYCP2*, *SYCE1*, *DMRTC2*, *ZPBP,* and *SPATA16* (Figure 2C,D, Supplementary Figure S1B–F). The cells in cluster 13 expressed the spermatid (SPT) marker genes *ZPBP*, *ACRV1*, *ACTL7B*, *HOOK1*, *TEX35*, *CAPZA3*, *HEMGN*, *TEX29,* and *SPATA19* (Figure 2D,E, Supplementary Figure S1G–M). The cells in cluster 18 expressed the SC marker genes *SOX9* and *WFDC2* (Figure 2F, Supplementary Figure S1N). The cells in clusters 6 and 15 expressed the EC marker genes *VWF*, *CD34,* and *PALMD* (Figure 2G, Supplementary Figure S1O,P). The cells in cluster 14 expressed the macrophage marker genes *C1QA* and *CSF1R* (Figure 2H, Supplementary Figure S1Q). The cells in clusters 4, 10, 11, and 16 expressed the LC/MC marker genes *CYP11A1*, *IGFBP5*, *INHBA*, *LGALS3*, *ACTA2*, *MYH11*, *TAGLN*, and *FHL2* (Figure 2I–K, Supplementary Figure S1R–V). The cells in clusters 1, 2, 7, 8, and 19 expressed the T-lymphocyte/natural killer cell (T/NK) marker gene *NKG7* (Figure 2L). This study successfully identified the main cell types in yak spermatogenesis (Figure 2M).

### 2.3. MCs and LCs Were Developed and Differentiated from the Same Progenitor Cells

**Figure 3:**
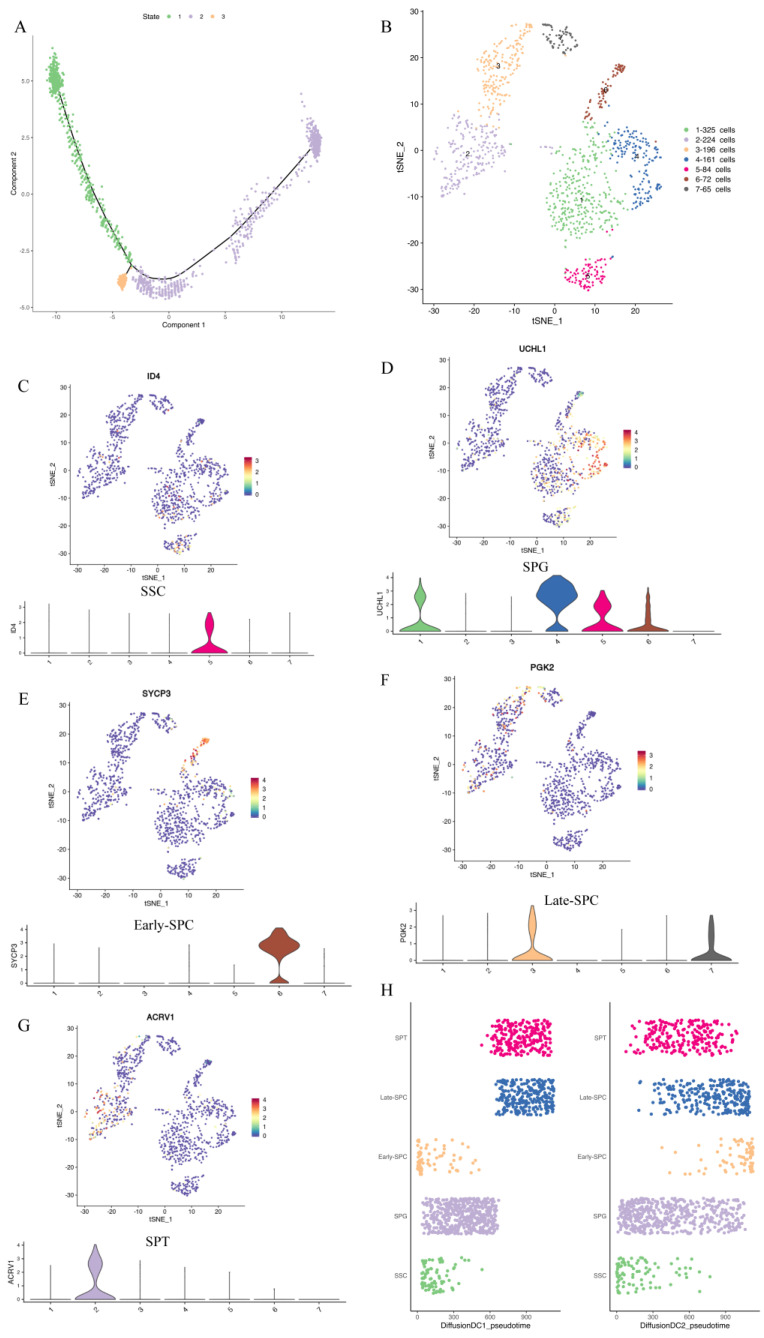
Pseudo-time analysis of LCs and MCs and re-clustering of germ cells. (**A**) Pseudo-time analysis of LCs and MCs. (**B**) The results of re-clustering of germ cells. Different clusters are represented by different colors. (**C**–**G**) The expression of marker genes in each cluster. (**H**) Sequencing results of cells in diffusion DC1–DC2.

Reduced dimension cluster analysis showed that LCs were located in clusters 4, 10, and 11, while MCs were found in clusters 10, 11, and 16. Guo et al. [31] studied adolescent human testis and found that LCs and MCs were developed from the same group of cells. To investigate whether MCs and LCs of yaks were derived from the same progenitor cells, we conducted a pseudo-time analysis of clusters 4, 10, 11, and 16. The Monocle pseudo-time analysis showed that LCs and MCs differentiated from common progenitor cells (Figure 3A).

### 2.4. Re-Clustering of Germ Cells

To study different types and subtypes of germ cells during yak spermatogenesis, we re-clustered (Figure 3B) germ cell populations (clusters 9, 12, 13, and 17 in Figure 2A) and determined the cell types based on genes expressed in each cell cluster (Supplementary Table S4) and the known marker genes. We identified SSCs as cluster 5 (Figure 3C, Supplementary Figure S2A), SPG as clusters 1 and 4 (Figure 3D, Supplementary Figure S2B), early–SPC as cluster 6 (Figure 3E, Supplementary Figure S2C–H), late–SPC as clusters 3 and 7 (Figure 3F, Supplementary Figure S2I–N), and SPT as cluster 2 (Figure 3G, Supplementary Figure S2N–Q). Moreover, we applied the DiffusionMap [34] to map the nonline-ar structure of the scRNA expression matrix based on the Gauss model [35] and the Mar-kov model [36] and found it was mapped to a continuous structure. We extracted the pre-three-dimensional eigenvalues and predicted the developmental state of each cell based on the sequence of eigenvalues, as shown in DC1–DC3 in Supplementary Table S5. Pseudotime1_diffusion represented the cell differentiation order obtained by sorting the cells in DC1 and the classification of each cell cluster and new cell type. Figure 3H shows the developmental trajectory of different cell types and subtypes (SSC–SPG–Early–SPC–Late–SPC–SPT) in different dimensions.

### 2.5. Identification of Marker Genes of Various Cell Types in Yak Testis

**Figure 4:**
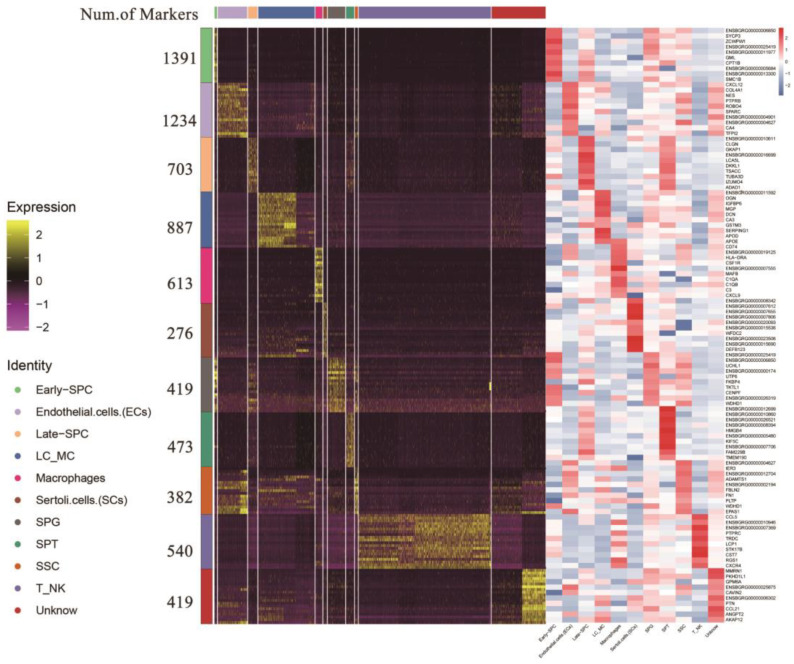
Heatmap of marker genes for each cell type. Different colors at the top of the heatmap represent different cell types. The right of the heatmap shows the number of marker genes and ID symbols of the top 10 markers. The color changes from purple to yellow and from blue to red show the expression levels from low to high.

Following identification, we integrated the clusters of the same yak testicular cell type (Supplementary Table S6). We identified 276 and 887 genes as marker genes in SCs and LCs/MCs, respectively. In male germ cells, we found 382, 419, 1391, 703, and 473 marker genes in SSC, SPG, early-SPC, late-SPC, and SPT, respectively. The top 10 gene IDs in each cell type are displayed on the right side of Figure 4, and most of them are either specifically expressed or highly expressed in the corresponding cell type (Supplementary Figure S3), including the classic marker genes *SYCP3*, *UCHL1*, and *TKTL1*, which provide potential marker genes for further study of testicular cell types.

### 2.6. Functional Enrichment Analysis of Yak Testicular Somatic Cells

**Figure 5:**
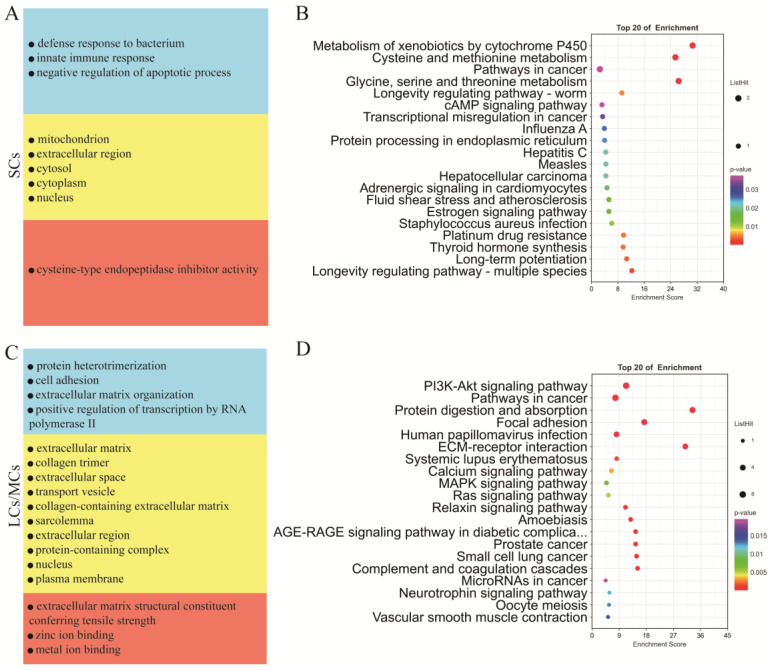
Functional enrichment analysis of different types of testicular somatic cells. (**A**) GO analysis of the top SC marker genes. (**B**) KEGG analysis of the top marker genes of SCs. (**C**) GO analysis of the top marker genes of LCs/MCs. (**D**) KEGG analysis of the top marker genes of LCs/MCs.

Testicular somatic cells are essential for testicular development and spermatogenesis. To investigate the distinct functions of Sertoli cells and LCs/MCs, we conducted GO and KEGG analyses (Supplementary Table S7) on their respective marker genes. Our GO analysis revealed that Sertoli cells were significantly enriched in the biological process category, including in the negative regulation of the apoptotic process, the defense response to bacteria, and the innate immune response; in the cellular component category, they were enriched in the mitochondrion, extracellular region, cytosol, cytoplasm, and nucleus; in the molecular function category, they were enriched in cysteine-type endopeptidase inhibitor activity (Figure 5A). Conversely, in the biological process category, LCs/MCs were significantly enriched in cell adhesion and extracellular matrix organization; in the cellular component category, they were enriched in the extracellular matrix, extracellular space, and extracellular region, and in the molecular function category, they were enriched in extracellular matrix structural constituents conferring tensile strength, and zinc ion binding (Figure 5C). KEGG analysis of the marker genes of SCs indicated significant enrichment in the cAMP signaling pathway, protein processing in the endoplasmic reticulum, and estrogen signaling pathway (Figure 5B). In contrast, marker genes of LCs/MCs were significantly enriched in the PI3K-Akt signaling pathway, MAPK signaling pathway, and ECM–receptor interaction (Figure 5D).

### 2.7. Functional Enrichment Analysis of Yak Testicular Germ Cells

**Figure 6:**
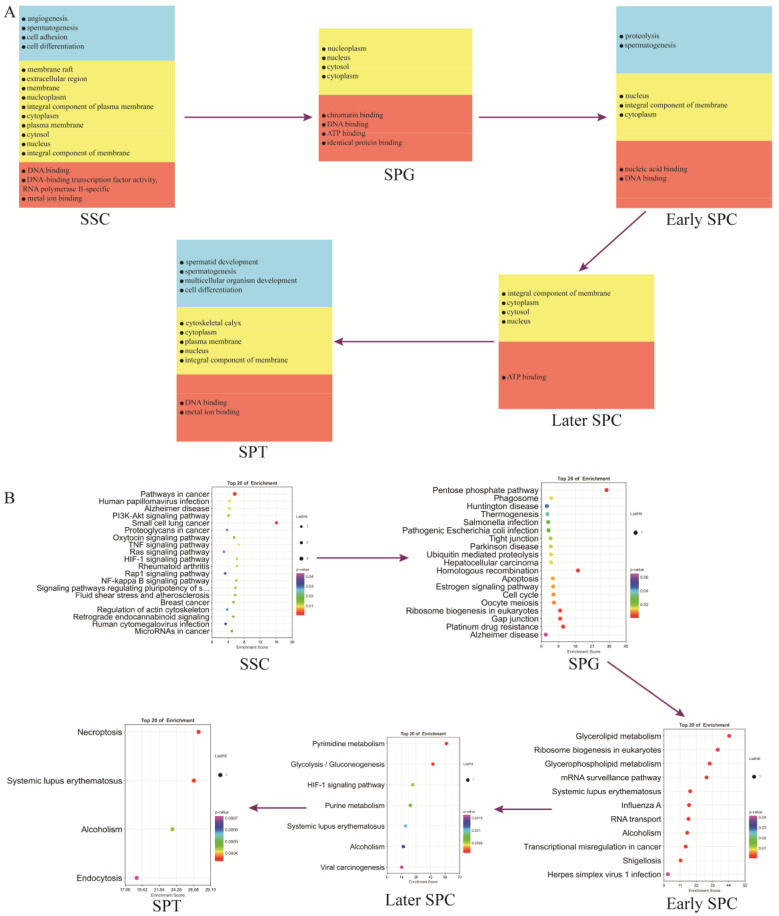
Functional enrichment analysis of testicular germ cell types. (**A**) GO analysis of the top marker genes of five germ cell types. (**B**) KEGG analysis of the top marker genes of five germ cell types.

Spermatogenesis involves intricate and sequential changes in spermatogenic cells. To investigate the differences between different germ cell types, we annotated the marker genes (Supplementary Table S8) of each germ cell cluster using the GO and KEGG analyses. Figure 6A illustrates that genes related to spermatogenesis, cell differentiation, DNA binding, ATP binding, and other functions were present from SSCs to SPT throughout the entire spermatogenesis process. The marker genes of SSCs were significantly enriched in several signaling pathways, such as those regulating the pluripotency of stem cells, PI3K-Akt signaling pathway, Ras signaling pathway, and actin cytoskeleton. Most of these genes were involved in spermatogenesis. The marker genes of SPG were significantly enriched in the gap junction, tight junction, estrogen signaling pathway, and cell cycle signaling pathways. The marker genes of early SPC were significantly enriched in ribosome biogenesis in eukaryotes, the mRNA surveillance pathway, and the RNA transport signaling pathway. The marker genes of late-SPC were significantly enriched in glycolysis/gluconeogenesis and the HIF-1 signaling pathway. The marker genes of SPT were mainly involved in necroptosis and the endocytosis signaling pathways (Figure 6B).

### 2.8. Validation of the Candidate Marker Genes of the Testis

**Figure 7:**
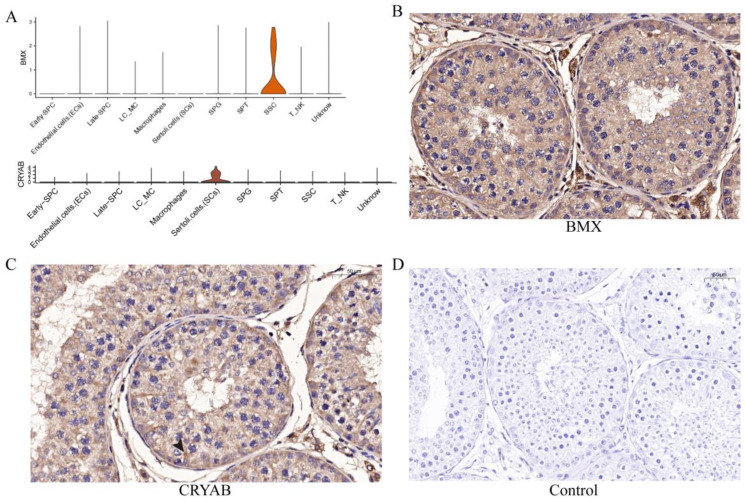
Expression and distribution of several potential genes. (**A**) The distribution of *BMX* and *CRYAB* expression in yak testicular cells. (**B**,**C**) Immunohistochemistry analysis of the location of BMX and CRYAB in yak testis. (**D**) Control group.

To further verify the sequencing results and identify the marker genes of each cell type, we selected several cell-specific expression genes (Figure 7A) in different cell types for immunohistochemical verification, including *BMX* in SSCs and *CRYAB* in SCs. The results showed that BMX was expressed in one circle of SSCs adjacent to seminiferous tubules (Figure 7B), while CRYAB was expressed in the supporting cells from seminiferous tubules to the seminiferous lumen (Figure 7C).

## 3. Discussion

Although the cell type composition of testicular tissue is similar across mammalian species and more than 80% of protein-coding genes are expressed in the testicular tissue of humans and other species [37,38], there are some differences in the testicular tissue of each species, such as varying differentiation times from SPG to mature sperm among human, mouse, and monkey and different SPG cycles between primates and mice [7]. Additionally, significant divergences exist in sex chromosome gene content and functional specialization, as evidenced by protein sequence changes and X chromosome gene acquisition [39,40].

In this study, we utilized scRNA-seq to investigate the heterogeneity of yak testicular tissue and identified cluster-specific expression of characteristic genes for each cluster, which enabled us to deduce the corresponding cell types. By analyzing 11,991 cells from sexually mature yak testes, we identified the main germ cell types and somatic cell types of yak testicles after sexual maturation. However, since there was a lack of information related to marker genes in each cell type of yak testicular tissue, we used cell markers from mice, humans, macaque, and sheep [7,8,9,10,28,30,31,32,41,42,43,44]. Our study not only clarified the cell types in yak testicular tissue after sexual maturation but also identified a series of candidate genes that may play crucial roles in spermatogenesis and provided marker genes for the further accurate study of yak testicular tissue cells.

Peritubular myoid cells are located at the periphery of the seminiferous tubules, providing contractile functions and secreting GDNF, a factor essential for spermatogenesis [31]. In contrast, LCs are located between the seminiferous tubules and secrete testosterone, which is crucial for maintaining normal spermatogenic processes, male secondary sexual characteristics, and systemic metabolism [45]. Yu et al. [44] and Guo et al. [31] carried out Monocle pseudo-time analyses on MCs and LCs of dairy goats and showed that the LCs and MCs differentiated from common progenitor cells. Guo et al. [31] found that the LCs and MCs were initially grouped into two separate clusters rather than a single large cluster. To explore whether this situation exists in yak testicles, we performed cluster analyses of yak testicular cells and found that yak LCs and MCs were grouped in a large cluster. Our Monocle pseudo-time analysis showed that LCs and MCs were differentiated from common progenitor cells (Figure 3A), consistent with the results reported by Guo et al. [31].

SCs, the only somatic cells in direct contact with germ cells, provide various “nursing functions” [46] for self-renewal, proliferation, and differentiation of SPG and spermatogenesis within the seminiferous tubules [47]. In addition, SCs are indispensable for the functional maintenance of adult testicular LCs and MCs [48], and the interaction among these cells is critical for controlling spermatogenesis [49]. To further study the role of testicular somatic cells in yak spermatogenesis, we performed a function enrichment analysis of the top genes of the major niche cell types, including SCs and MC/LCs. Zhang [50] et al. reported that marker genes in MCs were enriched in response to growth factor, extracellular matrix organization, and regulation of cell adhesion, that the marker genes in LCs were involved in testosterone synthesis, and that the marker genes in SCs were enriched in regulation of cell death and exocytosis. In a similar analysis of somatic cells in sheep testis, Yang et al. [10] found that SCs and LCs were mainly enriched in the ribosomal pathway, which is related to the function of protein molecules, likely due to higher ribosomal gene expression in sheep somatic cells. Our study revealed that SCs were mainly enriched in the negative regulation of apoptotic process and some metabolic pathways, protein processing in the endoplasmic reticulum, and in the cAMP signaling pathway, while LCs/MCs were mainly enriched in the PI3K-Akt signaling pathway, MAPK signaling pathway, and protein digestion and absorption, indicating the involvement of protein molecules in the function of yak testicular somatic cells. Although functional enrichment of testicular somatic cells differs slightly among species, their primary role is to assist spermatogenesis.

We initially targeted the large clusters of SPG, SPC, and SPT with known germ cell marker genes. A preliminary analysis of all the cells did not clearly distinguish the subtypes of germ cells at each stage, but re-clustering of the germ cells identified five types of yak germ cells. Further pseudo-time analysis of the dynamic development pattern of yak germ cells revealed that their development trajectory fully conforms to the known germ cell development track. We observed continuous expression of some genes, including *SYCP1* and *WDHD1* (also known as *CTF4)*, that were continuously expressed throughout the whole spermatogenesis process but specifically highly expressed at a certain stage. CTF4 is crucial for the cohesion of sister chromatids and regulation of the post-transcriptional step of the centromeric silencing pathway [51]. It also plays a role in DNA replication, a crucial step in cell division, and in multicellular biological development [52]. We also found a large number of genes that were specifically expressed at certain stages of spermatogenesis, such as *BMX* and *PTGS2* which were specifically expressed in SSC, while *SYCP3* was specifically expressed in early-SPC. Gene expression analysis allowed the identification of specifically expressed markers in different types of yak testis cells and provided a certain basis for identifying testis cells in the later stage. Moreover, we discovered a large number of specifically expressed genes that have not been studied or defined in each cell type, such as *ENSBGRG00000002194* in SSC, *ENSBGRG00000012408* in early-SPC, *ENSBGRG00000003672* in late-SPC, and *ENSBGRG00000013109* in SPT. These genes may play an important role in yak spermatogenesis. Our findings suggest that there may be many specific regulatory genes in the process of yak spermatogenesis, and further annotation and research are required.

Our study only identified five male germ cell types. However, we did not find any subtypes of SPG and SPT, in contrast to other studies [7,9,44]. This may be due to the small proportion of undiff-SPG in adult yak testis [53], leading to the limited number of cell types identified. Functional enrichment analysis of the top genes in various cell types revealed that the marker genes of SSCs were enriched in DNA-binding transcription factor activity and RNA polymerase II-specific activity, which is similar to the research on Angus bulls by Gao [54]. The KEGG database search also showed that the marker genes of SSCs were enriched in the signaling pathways regulating the pluripotency of stem cells, further validating the accuracy of cell typing based on the function of highly expressed genes. The marker genes of SPG were enriched in the cell cycle, consistent with the evidence that the proliferation ability of differentiated SPG is enhanced [55]. The marker genes of early- and late-SPC showed similar molecular function enrichment, suggesting that the marker genes of these two cell types were mainly related to meiosis.

Our study provides a systematical classification of testis cell groups in yaks based on previous studies on humans, mice, and sheep. We identified the main cell types, clarified their associated biological processes, and provided marker genes for future studies on their functions. We also provided detailed classification of yak germ cells and cell surface markers for studying SSC development in vitro. Our data set and analysis provide useful resources, references, and basic information for further genetic improvement of yaks. However, we acknowledge that our current work mainly focused on transcriptome analysis and protein validation with key markers. RNA expression does not always linearly reflect protein abundance, and we only analyzed a single testicle sample. Given the complexity of mammalian testicular development and the gradual process of puberty, future studies with more samples may reveal more details about developmental processes and transitions.

## 4. Materials and Methods

### 4.1. Ethics Statement

All animal-related procedures were conducted in accordance with the guidelines established by the China Council on Animal Care and the Ministry of Agriculture of the People’s Republic of China. All yak-handling procedures were approved by The Animal Care and Use Committee of the Lanzhou Institute of Husbandry and Pharmaceutical Sciences Chinese Academy of Agricultural Sciences (Permit No: SYXK-2014-0002).

### 4.2. Animals and Sample Collection

A healthy 4-year-old male yak from Xiahe County (34°51′50″ N, 102°26′9″ E) in Gannan Tibetan Autonomous Prefecture was selected for castration. The testicles were collected and washed with DPBS (Gibco, Waltham, MA, USA). The cleaned testicle was placed in a clean 90 mm petri dish and cut longitudinally with a scalpel. Tissues perpendicular to the long axis were cut into several 2–4 mm^3^ tissue blocks, placed in strain preservation tubes filled with 1 mL cryoprotectant (10% DMSO + 10% fetal bovine serum + 80% complete medium), and transported back to the laboratory for single cell separation within 24 h after gradient cooling.

### 4.3. Preparation of Single Testicular Cell Suspension

Frozen testicular tissue blocks were thawed by placing them immediately in a 37 °C water bath for 2 min. Afterward, the blocks were transferred with tweezers into resuscitation solution (DMEM/F12 medium + 0.5 mol/L sucrose + 20% FBS) in a 37 °C water bath for another 2 min. Then, the tissue blocks were washed with preheated DPBS 2–3 times, placed in DMEM/F12 medium (Gibco, Waltham, MA, USA), and cut into small pieces using ophthalmic scissors. The cut testicular tissues were placed into a 15 mL centrifuge tube containing a solution of 0.2% collagenase IV (2 mg/mL) (Sigma, City of Saint Louis, MO, USA) and a hyaluronidase (2 mg/mL) solution (Sigma, City of Saint Louis, USA) (1:1) of 8–10 times the total amount of the tissues, and incubated at 37 °C under shaking at 120 rpm for 30 min. After 15 min, 200 μL of tissue digestive fluid was taken every 3 min to observe the digestive condition under a microscope until free tubules with no mass tissues were clearly observed. The mixture was then centrifuged at 1000 r/min for 5 min, and the supernatant was drained. The tissue block was further digested with a 0.25% trypsin solution (2.5 mg/mL) (Gibco, Waltham, MA, USA) in a quantity of 8–10 times the total tissue block amount under at 37 °C shaking at 120 rpm for 40 min. After 15 min, 200 μL tissue digestible solution was taken every 3 min to observe the digestion condition under a microscope until single cell suspension was clearly observed. The digestion was then terminated by adding fetal bovine serum. The mixed cell suspension was blown even and filtered through 70 and 40 μm screens successively. The filtrate was collected and centrifuged at 300× *g* at 4 °C for 5 min. After the supernatant was discarded, the cells were re-suspended in DMEM/F12 complete medium containing 10% FBS and cultured at 37 °C with 5% CO_2_. Part of the cell suspension was stained with Trypan blue and counted using an automatic counter to calculate cell number and viability. Cells with viability >90% were adjusted to ~1000 cells/μL and used for scRNA seq.

### 4.4. Preparation and Sequencing of Testicular Single-Cell RNA Sequencing Library

The cell suspension was loaded onto the Chromium Single Cell Controller instrument (10× Genomics, Pleasanton, CA, USA). Cells with a Cell Barcode were encapsulated in droplets along with beads. The droplet-containing cells were collected, and the cells were lysed, allowing the mRNA in the cells to bind to the bead Cell Barcode and form single cell GEMs. A reverse transcription reaction was performed in the droplets to construct the cDNA library. GEM-RT-PCR was carried out with a 96-well reaction module (Bio-Rad; CT022510) in the C1000 Touch thermal cycler to generate barcoded cDNAs following these steps: 53 °C for 45 min; 85 °C for 5 min, and maintenance at 4 °C. The sample index on the library sequence was used to distinguish the source of the target sequence. The final library was sequenced on an Illumina Novaseq 6000. All data were deposited into the Gene Expression Omnibus (GEO) database (Accession number: GSE225618).

### 4.5. Quality Control, Mapping, and Clustering Analysis

Sample quality control was performed using the 10× genomics (v2.2.0) official software CellRanger v4.0 (https://www.10xgenomics.com/, accessed on 16 De-cember 2022), which employs STAR [56] to eliminate data from multi-cells, double cells and cells that are not combined. In this project, we considered cells with the number of retained cell genes and UMI within the range of mean ± 2 times the standard deviation and the proportion of mitochondrial genes less than 30% as high-quality cells for downstream analysis. To assess the quality of each sample, we mapped the reads to the reference genome version BosGru3.0 and obtained quality control results such as the number of high-quality cells, number of genes, and genome alignment rate in the original data. Principal component analysis (PCA) was carried out to reduce the dimensionality of the log-transformed gene-barcode matrix of the top highly variable genes onto a smaller set of composite variables using the function “RunPCA”. The top 2 principal components (PCs) and their compositions were visualized by the functions “DimPlot” and “VizDimLoadings,” respectively. We used principal component analysis (PCA) and t-distributed stochastic neighbor embedding (t-SNE) algorithms for dimension reduction clustering analysis and visualized the PCA-based dimension reduction results using t-SNE for single cell cluster clustering. K-means clustering was used to cluster cells across a range of K values (2–30), where K is the preset number of clusters. The default selected value of K is that which yields the best Davies–Bouldin Index, a rough measure of clustering quality. We employed SNN as the clustering algorithm to obtain the optimal cell cluster and identified cell clusters based on reported marker genes (Supplementary Table S9).

### 4.6. Top Expressed Genes and Functional Enrichment Analysis

GO and KEGG enrichment analyses of top expressed genes were performed by using the methods implemented in the GOseq R and KOBAS 3.0 package, respectively. Top expressed genes in each cluster were screened according to the FoldChange and differential significance test (*p*-value) results. The function of top expressed genes identified from different germ and niche cell cohorts were primarily analyzed by functional enrichments and predicted by published data.

### 4.7. Cell Trajectory Analysis

Cell trajectory analysis, also known as pseudo-time analysis, is a useful approach for revealing the differentiation trajectory of cells or the evolution of cell subtypes during the development process. The method involves sorting cells at pseudo-time based on the expression patterns of key genes, which allows for the simulation of dynamic changes occurring during the development process. Thus, we performed cell trajectory analysis of male germ cells, LCs, and MCs using the Monocle 2 package (v2.8.0).

### 4.8. Histological Observation and Immunohistochemical Analysis

Testicular tissue was sectioned, stained, and immunohistochemically verified using previously established methods [57,58]. The tissue was rinsed with running saline and then cut into small pieces with a size of about 5 mm^3^ using a sterile scalpel. The testicular block was immediately rinsed with DPBS and fixed in animal testicular tissue fixative (Servicebio, Wuhan, China) at room temperature for 20–24 h, with fixative replacement at the 6th and 12th hour of fixation. Following fixation, the testicular block was transferred to 75% ethanol for preservation or for the subsequent sectioning and hematoxylin-eosin staining. Immunohistochemical analysis was conducted to identify the expression and localization of several marker genes using the rabbit anti-alpha B crystallin antibody (1:100 dilution, bs-4651R; Boosen, Beijing, China) and rabbit anti-BMX antibody (1:100 dilution, bs-2765R; Boosen, Beijing, China). Goat anti-rabbit IgG (1:100 dilution, SP-9001; Jin Qiao, Beijing, China) was used to catalyze the conversion of DAB to a brown precipitate during immunohistochemical staining.

## 5. Conclusions

We identified 11 distinct cell types in yak testis using scRNA-seq. Among them, five were germ cells, including SSC, SPG, early-SPC, late-SPC, and SPT, and six were somatic cells, including SCs, LCs, MCs, ECs, macrophages, and T/NK cells. We obtained specific expression or high expression genes for each cell type and performed functional enrichment analysis, which identified candidate marker genes for further investigation of testicular cell function. Our findings suggest that yak LCs and MCs are derived from a common progenitor cell. We also identified *BMX* and *CRYAB* as marker genes for SSCs and SCs using immunohistochemistry. Our dataset provides valuable insights into yak spermatogenesis and paves the way for identifying key molecular markers involved in male germ cell development.

## Data Availability

The raw reads of scRNA–seq of yak testis are available at GEO under the accession number GSE225618.

## Supplementary Materials

The following supporting information can be downloaded at: https://www.mdpi.com/article/10.3390/ijms24097982/s1.
