# Supplementary figures and images for: Single-Cell RNA Sequencing Reveals Atlas of Yak Testis Cells
Source: Int J Mol Sci. 2023 Apr 28;24(9):7982. doi: 10.3390/ijms24097982 (PMC10178277; doi:10.3390/ijms24097982)

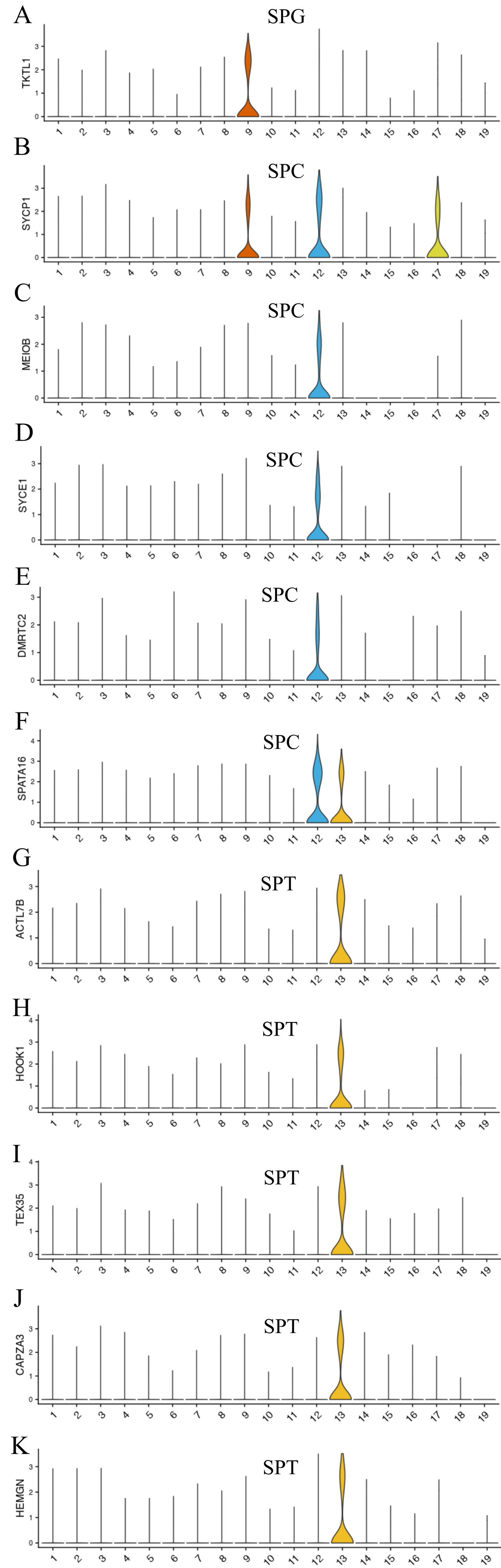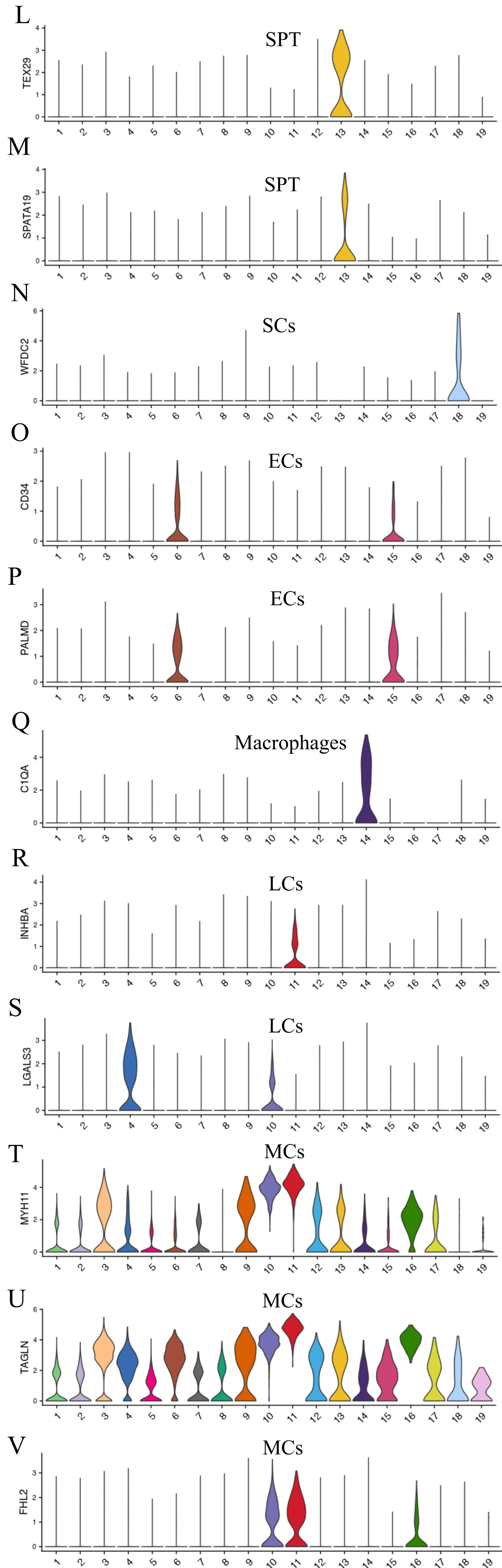

Supplement: Supplementary file 1 [file ijms-24-07982-s001.zip › Supplementary Figure S1.pdf]

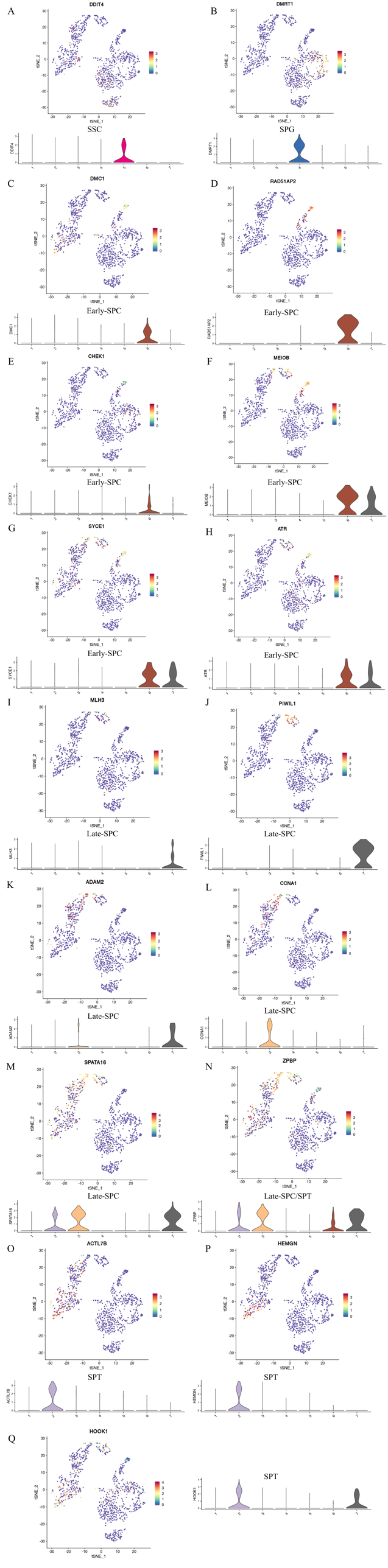

Supplement: Supplementary file 1 [file ijms-24-07982-s001.zip › Supplementary Figure S2.pdf]
