# Supplementary material for: Single-Cell RNA Sequencing Reveals Atlas of Yak Testis Cells
Source: Int J Mol Sci. 2023 Apr 28;24(9):7982. doi: 10.3390/ijms24097982 (PMC10178277; doi:10.3390/ijms24097982)
Supplement: Supplementary file 1 [file Supplementary Figure S3.pdf › Supplementary Figure S3.pdf]

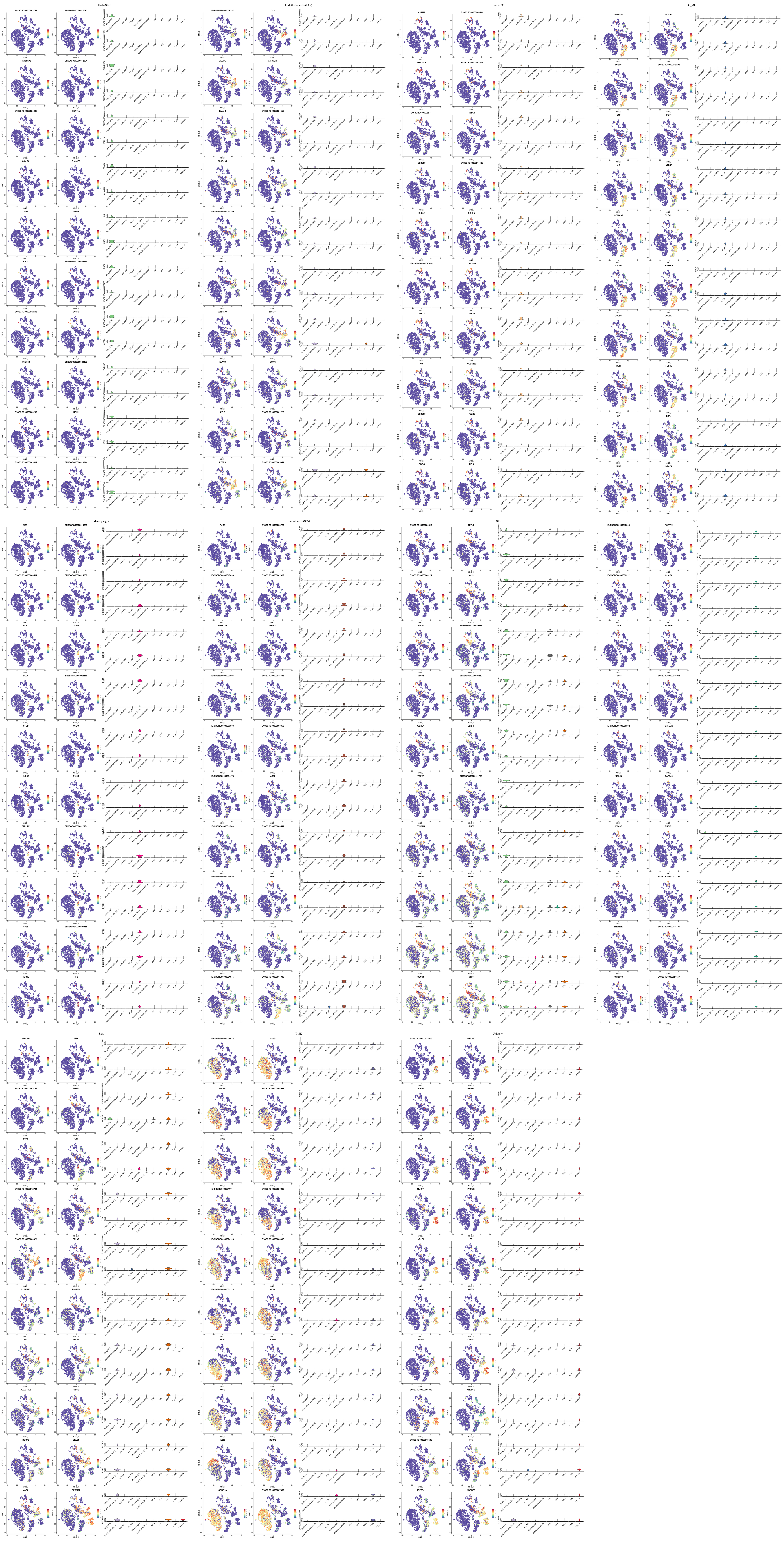
Early-SPC
Endothelial cells (ECs)
Late-SPC
LC_MC
Macrophages
Sertoli cells (SCs)
SPG
SPT
SSC
T-NK
Unknow
